# “And we feel success” – Participant reflections of a multi‐component group reablement program for people with dementia and their care partners

**DOI:** 10.1002/alz70858_105401

**Published:** 2025-12-26

**Authors:** Georgina Chelberg, Diane Gibson, Lara Wiseman, Helen Holloway, Kasia Bail, Stephen Isbel, Rachael Mitterfellner, Michelle Bennett, Nathan M D'Cunha

**Affiliations:** ^1^ University of Canberra, Bruce, ACT, Australia; ^2^ Canberra Health Services, Bruce, ACT, Australia

## Abstract

**Background:**

Australia's National Dementia Action Plan 2024‐2034 highlights the challenging post‐diagnostic journey often faced by people with dementia and care partners. A novel multidisciplinary, multicomponent program was designed to address this gap as an outpatient clinic at a rehabilitation hospital. The ‘SPICE Program’ (Sustainable Personalised Interventions for Cognition, Care, and Engagement) is delivered in small groups and individual appointments by allied health professionals over twelve‐weeks. Program components include Cognitive Stimulation Therapy, the Care Of People with Dementia (COPE®) Program, care partner education, exercise and dietetics. This study presents analysis of the experiences and perspectives of participants who completed SPICE during 2022‐2024.

**Method:**

Semi‐structured interviews were audio‐recorded after program completion. Transcripts were grouped and underwent content analysis by four researchers to identify codes and themes driven from a line of enquiry that prompted for self‐reported changes and program feedback.

**Result:**

Participants for each of the first ten SPICE Programs included up to seven people with dementia (*n* = 60, M=79yrs; 42% female) and their care partners (*n* = 58; M=75yrs; 67%female) living in the community. People with dementia shared themes of SPICE being ‘stimulating and useful’ with social ‘connections’, ‘enjoyment’ and increased ‘confidence’ through new friendships with peers and staff. Care partners reported positive changes in their loved one with similar themes of ‘connection’, ‘enjoyment’, ‘being understood’, as well as enhancements in physical and social capacity. Self‐reflection by care partners involved themes of ‘belonging’, ‘sharing and learning together', increased ‘understanding’ and caring ‘breakthroughs’.

**Conclusion:**

Self‐reported and proxy‐reflections on the SPICE program highlight the positive experiences and wellbeing impacts for a majority of participants. The group context offered important opportunities for social connection for participants, acting as a catalyst that enhanced potential benefits in other domains of well‐being. Findings extend the evidence for wider availability of early post‐diagnostic care in Australia with further research needed to develop implementation guidelines for comparable programs.

